# Fusion protein based strategies for developing insect-resistant crops

**DOI:** 10.1007/s10142-026-01861-9

**Published:** 2026-03-31

**Authors:** Marri Keerthana, Morthala Shankara Sai Reddy, Yogesh Dashrath Naik, Arindam Pal, Anil Namdev Kale, Satish Kumar Singh, Sudha Nandni, K. Premalatha, Murukarthick Jayakodi, Venugopal Mendu, Manish K. Pandey, Somashekhar Punnuri, Rajeev K. Varshney, Mahendar Thudi

**Affiliations:** 1 PG College of Agriculture, Dr. Rajendra Prasad Central Agricultural University, Pusa, Samastipur, Bihar 848125 India; 2 College of Basic Science and Humanities, Dr. Rajendra Prasad Central Agricultural University, Pusa, Samastipur, Bihar 848125 India; 3 Agricultural Science Center, New Mexico State University, Clovis, NM 88101 United States; 4 Institute of Agricultural Sciences, Banaras Hindu University, Varanasi, Uttar Pradesh 221005 India; 5 Department of Agricultural Entomology, Tamil Nadu Agricultural University, Coimbatore, 641 003 India; 6 Texas A&M AgriLife Research Center, Dallas, TX USA; 7 Department of Soil & Crop Sciences, Texas A&M University, College Station, TX USA; 8 Agriculture, Agribusiness and Environmental Sciences, Texas A&M University, Kingsville, USA; 9 International Crops Research Institute for the Semi-Arid Tropics, Hyderabad, Telangana India; 10 College of Agriculture, Family Sciences and Technology, 1005 State University Dr, Fort Valley State University, Fort Valley, GA USA; 11 WA State Agricultural Biotechnology Centre, Centre for Crop and Food Innovation, Murdoch University, Murdoch, WA 6150 USA; 12 Institute for Agriculture, Climate and Environment, University of Southern Queensland, Toowoomba, Australia

**Keywords:** Fusion proteins, Insect resistance, Transgenic crops, Single gene constructs, Scorpion toxins, Spider venom

## Abstract

Insect pest control in crop production incurs substantial economic costs annually on a global scale. Although broad-spectrum chemical pesticides were once considered the most effective solution, their overreliance has led to adverse effects on beneficial insects, human health, and the environment, as well as the development of pesticide-resistant insect populations. Consequently, there is an urgent need for alternative pest management strategies that minimize pesticide use and reduce unintended impacts on natural enemies, thereby maintaining ecological balance. Host plant resistance plays a pivotal role in integrated pest management (IPM). However, developing pest-resistant varieties through conventional breeding methods can be time-consuming and challenging due to the involvement of multiple quantitative traits controlled by various genetic loci. One promising biotechnological approach is the development of fusion proteins, engineered molecules that combine the functional properties of two or more distinct proteins. These fusion proteins effectively target specific insect pests while minimizing environmental impact. Importantly, they overcome key limitations of single-gene constructs, including narrow target range and rapid resistance development. Numerous fusion proteins have been successfully developed and deployed in various crop plants, demonstrating their versatility and broad-spectrum activity against major insect pests. This review discusses fusion protein technologies and their application in developing transgenic crops with enhanced resistance to insect pests.

## Introduction

Recent research indicates that the world’s population is expected to grow by 2 billion over the next three decades, reaching approximately 10 billion by 2050 (Zsögön et al. 2022). This rapid population growth is projected to substantially escalate food demand, with an anticipated 56% surge from 2010 levels by 2050 (Van Dijk et al. 2021). To meet these growing demands, enhancing crop yields and minimizing pre- and post-harvest losses are crucial. Beyond the unprecedented rise in population, the depletion of natural resources, climate change, and the emergence of insect pests and disease-causing pathogens pose serious constraints on crop production and productivity (Alemu 2020).

Insect pests are a major constraint to global agricultural productivity, causing substantial yield losses and significant economic damage across diverse cropping systems. They are estimated to account for approximately 25–30% of global agricultural losses (Joshi et al. 2020; Mateos Fernández et al. 2022). Several destructive pests exert particularly severe impacts, including the fall armyworm (*Spodoptera frugiperda*) affecting maize and cereals across Africa, Asia, and the Americas (Matova et al. 2020); the desert locust (*Schistocerca gregaria*) devastating crops in Africa, the Middle East, and South Asia (Mitra et al. 2024); the brown planthopper (*Nilaparvata lugens*) reducing rice yields (Zheng et al. 2021); stem borers (*Chilo partellus*, *Busseola fusca*) weakening maize and sorghum plants (Benjamin et al. 2024); and the Colorado potato beetle (*Leptinotarsa decemlineata*), which threatens potato production due to rapid resistance development (Chen et al. 2023). In pulse crops, *Helicoverpa armigera* causes significant losses in chickpea and related species (Reddy et al. 2022). In addition to food crops, this pest has caused devastating damage to cotton production in China, where large-scale adoption of Bt cotton resulted in marked suppression of *H. armigera* populations across multiple crops, clearly demonstrating the economic and ecological benefits of transgenic pest-resistant technologies (Wu et al. 2008).

To mitigate such pest pressure, conventional pest management has relied heavily on chemical insecticides. However, excessive and prolonged use of these chemicals has resulted in serious limitations, including environmental contamination, risks to human health, adverse effects on biodiversity and pollinators, and the rapid evolution of resistance in insect pest populations. These drawbacks highlight the unsustainability of chemical control as a standalone strategy. Consequently, integrated pest management (IPM) has emerged as a rational and environmentally responsible approach, emphasizing reduced chemical dependence through the integration of biological control, host plant resistance, and advanced biotechnological interventions to achieve durable and sustainable pest suppression. The global insecticides market was valued at USD 9.12 billion in 2022 and is expected to grow at a rate of 4.7% annually (Polaris Market Research 2024). Fungi and viruses are recognized as the most significant plant pathogens, becoming more prevalent and severe, posing a major threat to agriculture due to the lack of suitable agrochemicals and the absence of resistance and/or immunity in host plants. Therefore, the necessity of developing advanced eco-friendly strategies for pest management is becoming a top priority (Pathak et al. 2022). Host plant resistance (HPR), an environmentally friendly alternative to chemical control, offers the potential for sustainable pest management, with the challenge of developing resistant varieties to enhance crop productivity in a rapidly changing world (Wani et al. 2022).

The utilization of a new era of microbial biopesticides, i.e., microorganisms are used for their properties to produce toxins or antimicrobial proteins, has shown a consistent annual growth of 10%, with over 225 microbial biopesticides authorized in 30 nations within the Organization for Economic Co-operation and Development (OECD) (Kabaluk and Gazdik 2007). Additionally, it is estimated that the market size of microbial pesticides is USD 1.37 billion in 2024 and expected to increase to USD 1.87 billion by 2029 (Mordor Intelligence 2024). Among microbial biopesticides, *B. thuringiensis* (Bt) is the most widely used and commercially successful biological insecticide, accounting for the largest market share and economic returns due to its high specificity, safety to non-target organisms, and proven effectiveness against major lepidopteran, coleopteran, and dipteran pests. Bt-based formulations and transgenic Bt crops have been extensively integrated into pest management programs worldwide, highlighting their central role in modern biological control (Bravo et al. 2011). Among these approaches, antimicrobial peptides have emerged as promising biopesticidal agents, and recent advances in understanding their modes of action and potential agricultural applications have renewed interest in their use for insect pest management (Dho et al. 2023). While conventional insecticides and microbial biopesticides currently dominate the crop protection market, fusion protein–based technologies represent an emerging biotechnological approach aimed at improving target specificity and resistance durability. Although still largely at the research and regulatory evaluation stage, increasing interest in biologically derived and multi-functional pest control strategies suggests strong potential for future adoption of fusion protein technologies in transgenic crop systems.

During the last two decades, several studies have advanced our understanding of the genetic mechanisms of insect pest resistance in crop plants (Du et al. 2020; Arif et al. 2022; Yan et al. 2023; Rehman et al. 2024). Various genetic loci associated with insect pest resistance have been identified and used in crop breeding programs for developing resistant varieties (Luo et al. 2023; Miah et al. 2013). Further unconventional routes to develop durable insect resistance were also explored (Enders and Begcy 2021). While Marker Assisted Selection (MAS) remains a valuable tool for developing insect-resistant crops, fusion protein technology offers superior precision, speed, and broader resistance. By enabling the development of multi-functional proteins with enhanced stability and efficacy, fusion protein technology provides a more direct and powerful approach to insect resistance management.

Fusion proteins represent a powerful molecular engineering strategy where two or more distinct functional domains are genetically linked into a single polypeptide (Chen et al. 2013). This technology is leveraged to overcome inherent limitations of single-component biopesticides, such as low stability, limited host range, or inefficient uptake (Sainsbury et al. 2012). In agricultural applications, these fusion proteins can be designed to effectively target specific pests or pathogens while minimizing environmental impact. Fusion proteins have also shown promising applications in nematode management and enhancing abiotic stress tolerance in plants. The development of fusion proteins involves the meticulous engineering of protein sequences to combine desired functionalities. Specifically, fusions are designed to enhance oral bioavailability (e.g., via gut-targeting domains like lectins), increase insecticidal potency or expand host range (e.g., synergistic toxin fusions), or improve protein stability and production yield (Ahmad et al. 2015). Furthermore, fusion protein technology offers the potential for sustainable and environmentally responsible pest and pathogen management strategies. Implementing fusion proteins as potential biopesticides can become an alternative to conventional chemical pesticides, reducing environmental load and opening a new era for transgenic pest control.

Fusion proteins for insect resistance can be classified based on their origin, mechanism of action, targeted insect groups, and structural design. Based on origin, they can be naturally occurring, such as those derived from bacteria or plants with inherent insecticidal properties or synthetically engineered using recombinant DNA technology to enhance their efficacy and specificity (Javaid et al. 2018). Mechanistically, fusion proteins function through various modes, including toxin-based approaches like Bt (*B. thuringiensis*) fusion proteins (e.g., Cry1Ac-Cry2Ab, Vip3A-Cry1Ab) that bind to insect gut receptors, leading to cell lysis (Bravo et al. 2011; Sainsbury et al. 2012); chitinase-based fusion proteins that degrade the insect exoskeleton and gut lining; protease inhibitor-based fusions that prevent insect adaptation to Bt toxins (Fitches et al. 2004); lectin-based fusions that interfere with digestion; and RNA interference (RNAi)-based fusion proteins that silence essential insect genes upon ingestion (Liu et al. 2025). Based on target specificity, fusion proteins can be tailored for lepidopteran pests (e.g., Cry1Ac-Cry2Ab targeting *H. armigera*) (Sainsbury et al. 2012), coleopteran pests (e.g., Cry3Bb1-Cry8Da targeting corn rootworm) (Bravo et al. 2011), hemipteran pests (e.g., lectin-based fusions for aphids and whiteflies) (Sukiran et al. 2023), or designed as broad-spectrum insecticidal proteins combining multiple mechanisms (Liu et al. 2016a). Structurally, fusion proteins can exist as linear fusions where toxins are directly linked, multi-domain proteins that enhance specificity and binding, or hybrid constructs that integrate synergistic toxin-inhibitor combinations for improved insecticidal activity (Javaid et al. 2018). These fusion proteins offer a promising solution for managing resistant insect populations and enhancing crop protection.

In this review, we highlight various fusion protein technologies being deployed for developing insect pest-resistant crop plants as well as comprehensively discuss the core strengths and weaknesses of these approaches. We explore the rapidly evolving landscape of fusion protein technology for insect pest management, examining various fusion proteins from Cry-based constructs to those incorporating lectins, scorpion toxins, spider venoms, and hormone disruptors. We further discuss their applications beyond insect control, addressing plant diseases, nematode infestations, and abiotic stress tolerance. Through critical analysis of their advantages, limitations, and prospects, we provide insights into how fusion protein technology could contribute to sustainable agricultural systems in an era of increasing pest pressure and environmental challenges. Collectively, these statistics and case studies underscore how advances in molecular engineering and transgenic technologies, particularly fusion protein design, translate fundamental biological knowledge into practical biotechnological solutions for durable and targeted pest management.

### Single gene constructs for insect resistance: scope and challenges

Single gene construct technology has emerged as a powerful approach for developing insect-resistant crop plants by introducing a single gene encoding an insecticidal protein. This technology enables precise and targeted pest control through various mechanisms. For instance, Bt crops such as Bt cotton, Bt maize, and Bt brinjal express *Cry* and *Vip* proteins from *B. thuringiensis*, which bind to insect gut receptors, causing cell lysis and mortality (Tabashnik and Carrière 2017). Similarly, RNAi-based crops, such as *SmartStax PRO* maize, employ the *DvSnf7* gene to silence essential insect genes, disrupting metabolism and leading to pest mortality (Baum et al. 2007). Additionally, proteinase inhibitor genes, such as the cowpea trypsin inhibitor gene, have been introduced into crops like tobacco and rice to inhibit insect digestive enzymes, causing starvation (Shelton et al. 2002). The advantages of single gene construct technology include high specificity, stable resistance, reduced environmental impact, and cost-effectiveness. However, single gene constructs in biotechnology and agriculture have often encountered significant limitations, leading to their failure in providing effective and sustainable solutions. Key issues include limited efficacy, as these constructs typically target specific pathogens or pests, making them insufficient against a wide range of threats. For instance, while many crops, such as cotton, maize, eggplant, tobacco, potato, rice, and tomato, have been genetically transformed with Bt genes, only cotton and maize have been commercially successful in achieving insecticidal activity. Bt toxins are effective against insects in the Lepidoptera and Coleoptera families (Heckel 2020). However, their specificity limits their effectiveness against many sucking pests, such as African rice green hoppers, rice thrips, white-backed bugs, and green leafhoppers (Javaid et al. 2018). Additionally, pests and pathogens can rapidly develop resistance to single gene constructs, reducing their long-term effectiveness. By contrast, fusion proteins are specifically engineered to overcome these limitations by combining multiple functional domains into a single molecule. This approach directly implements the strategies resistance, thereby providing a more robust and sustainable solution (Sainsbury et al. 2012). Despite significant advances in chemical control, microbial biopesticides, and host plant resistance, existing pest management strategies remain constrained by limited target range, resistance evolution and sustainability concerns. Single-gene transgenic approaches have provided effective but often narrow-spectrum protection, highlighting the need for integrated molecular strategies capable of delivering broader and more durable resistance. In this context, fusion protein technology represents a rational extension of current biotechnological approaches, enabling the combination of complementary functional domains within a single genetic construct to enhance efficacy, durability, and adaptability in insect-resistant crop development.

## Fusion proteins: rational design, mechanisms, and engineering

### Core strategies and advantages of fusion proteins

By combining multiple functional domains within a single genetic construct, fusion proteins enable coordinated activity against diverse insect targets while maintaining stable inheritance from a single locus. This integrated design inherently implements a pyramiding strategy, creating a higher genetic barrier to resistance evolution and enhancing the durability of insect resistance traits. As a result, fusion protein approaches provide a strategic framework for developing more robust and sustainable insect-resistant crops, forming the conceptual basis for the mechanistic and design-focused discussions presented in the following sections.

### Fusion Proteins and their mechanism

**Fig. 1:**
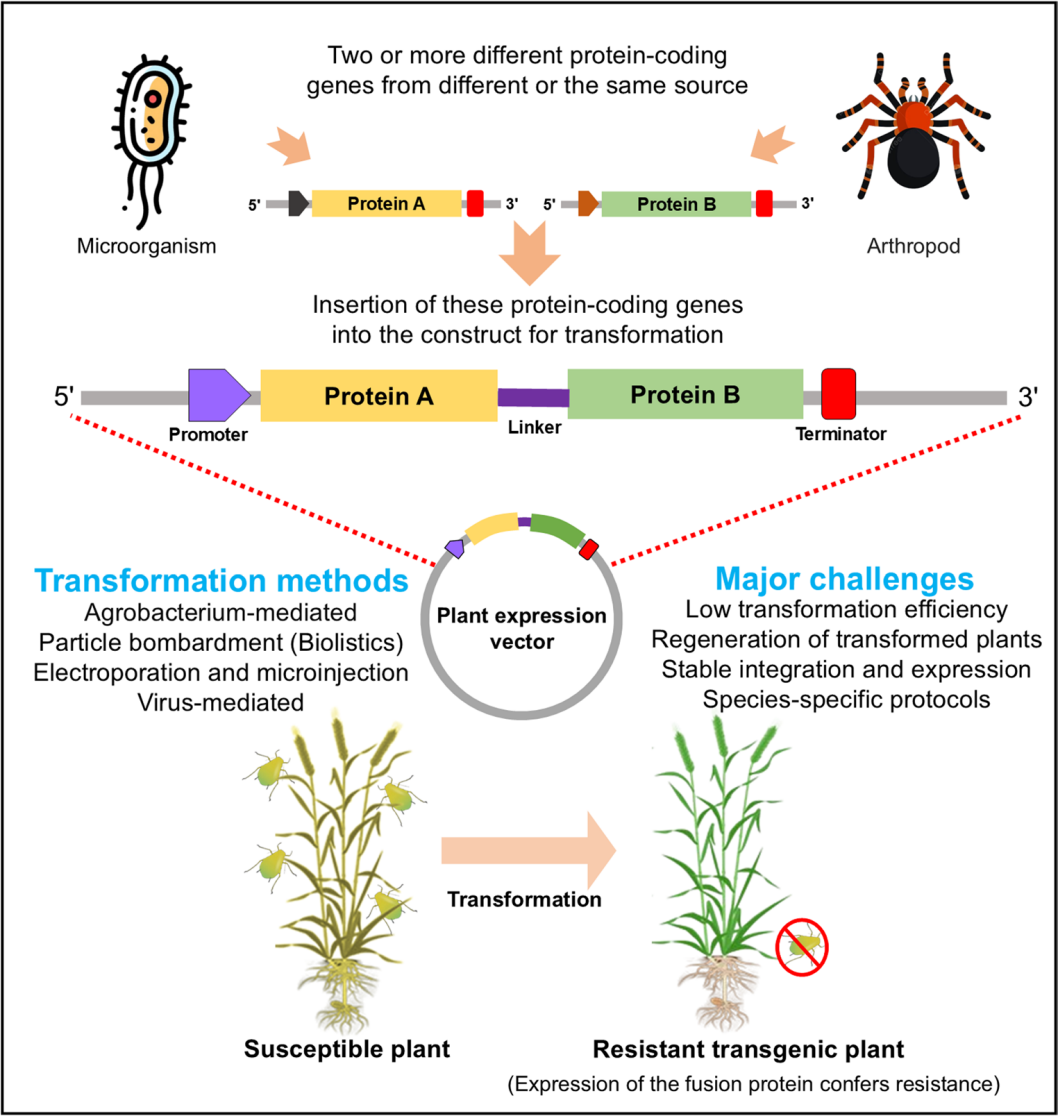
Insect resistance engineering through fusion protein transformation in plants. The schematic illustrates the design of a fusion protein construct by linking two protein-coding genes from the same or different sources under a promoter and terminator. The construct is introduced into plants using various transformation methods, leading to stable expression of fusion proteins that confer enhanced resistance to insect pests, while highlighting key challenges associated with plant transformation

Fusion proteins are widely used in molecular biology for both fundamental and applied studies. They are generated by combining two or more genes encoding distinct proteins or peptides under a common promoter and terminator, allowing for the coordinated expression of a single recombinant fusion protein in plants (Fig. 1). Initially, reporter proteins such as GUS, LUC and GFP were fused with target proteins to generate fusion proteins that facilitate the identification and monitoring of gene expression in transformed tissues (Khan et al. 2017; Súnico et al. 2024; Wang et al. 2024). More recently, advanced fusion proteins comprising two or more proteins derived from the same or different species have been developed for the effective control of diverse insect and pest species (Boddupally et al. 2018; Xu et al. 2018).

During the crop life cycle, insects such as Lepidopterans, Coleoptera, Hymenoptera, Diptera, Hemipterans, and others damage crops in various ways. The introduction of a single insecticidal gene using recombinant DNA technology generally confers resistance against a limited range of insect pests. Infection of the transgenic plant by another insect that has no resistance gene will result in reduced crop productivity and quality. In contrast, the fusion protein generated from *Cry1Ac* and *ASAL* from *B. thuringiensis* and garlic (*Allium sativum* L.) protects rice (*Oryza sativa* L.) against both lepidopteran and hemipteran insects, helping to improve rice yield (Boddupally et al. 2018).

The efficacy of fusion protein–based insect resistance depends on promoter selection, expression vectors, and fusion design. Constitutive promoters such as *CaMV35S* enable high expression but may increase metabolic burden or non-target exposure, whereas tissue-specific or inducible promoters provide spatial and temporal control (Potenza et al. 2004; Venter 2007). Several promoters, such as *PActin1*, *CaMV35S*, maize proteinase inhibitor, ubiquitin, and polyubiquitin-1, have been utilized to express fused proteins for insect resistance, with *CaMV35S* being the most frequently used promoter. Effective expression vectors must support stable transcription and proper folding of fusion constructs in plants. Fusion design parameters, including linker length and domain orientation, are critical for preserving activity and synergistic function (Chen et al. 2010). Compared with multi-gene stacking, fusion proteins allow coordinated expression from a single locus, reducing segregation effects and simplifying regulatory assessment (Sainsbury et al. 2012). Various fusion proteins have been developed to manage insects and pests (Xu et al. 2018; Majumder et al. 2020). These fusion proteins target insect voltage gated sodium and calcium ion channels to reduce insect populations on plants. The generation of a fusion protein relies on recombinant DNA technology to physically link the genes of interest. This is typically achieved by fusing the open reading frames, often via a synthetic linker sequence, to create a single continuous gene et al. 2013, Javaid et al. 2018). Selection of the constituent proteins or protein coding genes is a critical consideration and is generally based on the intended function of the fusion protein. The component genes can be derived from the same species (e.g., two different Cry genes from *B. thuringiensis* (Héma et al. 2009) or from phylogenetically distant organisms (e.g., a spider toxin fused to a plant lectin (Nakasu et al. 2014), highlighting the versatility of this platform. In the context of Bt-cotton, two distinct genes, *cry1AC* and *cry2AB*, were employed from a single microbe, *B. thuringiensis*, to control cotton bollworms and cotton leaf rollers (Héma et al. 2009). To suppress the yellow stem borer, (*Scirpophaga incertulas*), leaf folder (*Cnaphalocrosis medinalis*), and brown plant hopper (*N. lugens*), a fusion protein made by combining the Bt*Cry1Ac* gene from *B. thuringiensis* and carbohydrate-binding domain, *ASAL* from garlic (*Allium sativum*) has been introduced into rice (Boddupally et al. 2018). Some fusion proteins have also been produced from venoms extracted from various arachnids. For example, aphids were effectively controlled by combining the spiders derived *ω-ACTX-Hv1a* gene with the plant-derived *hwtx-I* gene (Nakasu et al. 2014). Some plant-based inhibitors are also employed to control insects. For instance, maize proteinase inhibitor (MPI) and potato carboxypeptidase inhibitor (PCI) have been used to control the striped stem borer (*Chilo suppressalis*) in rice (Quilis et al. 2014).

### Optimizing protein selection: functional, stability and expression factors

**Fig. 2:**
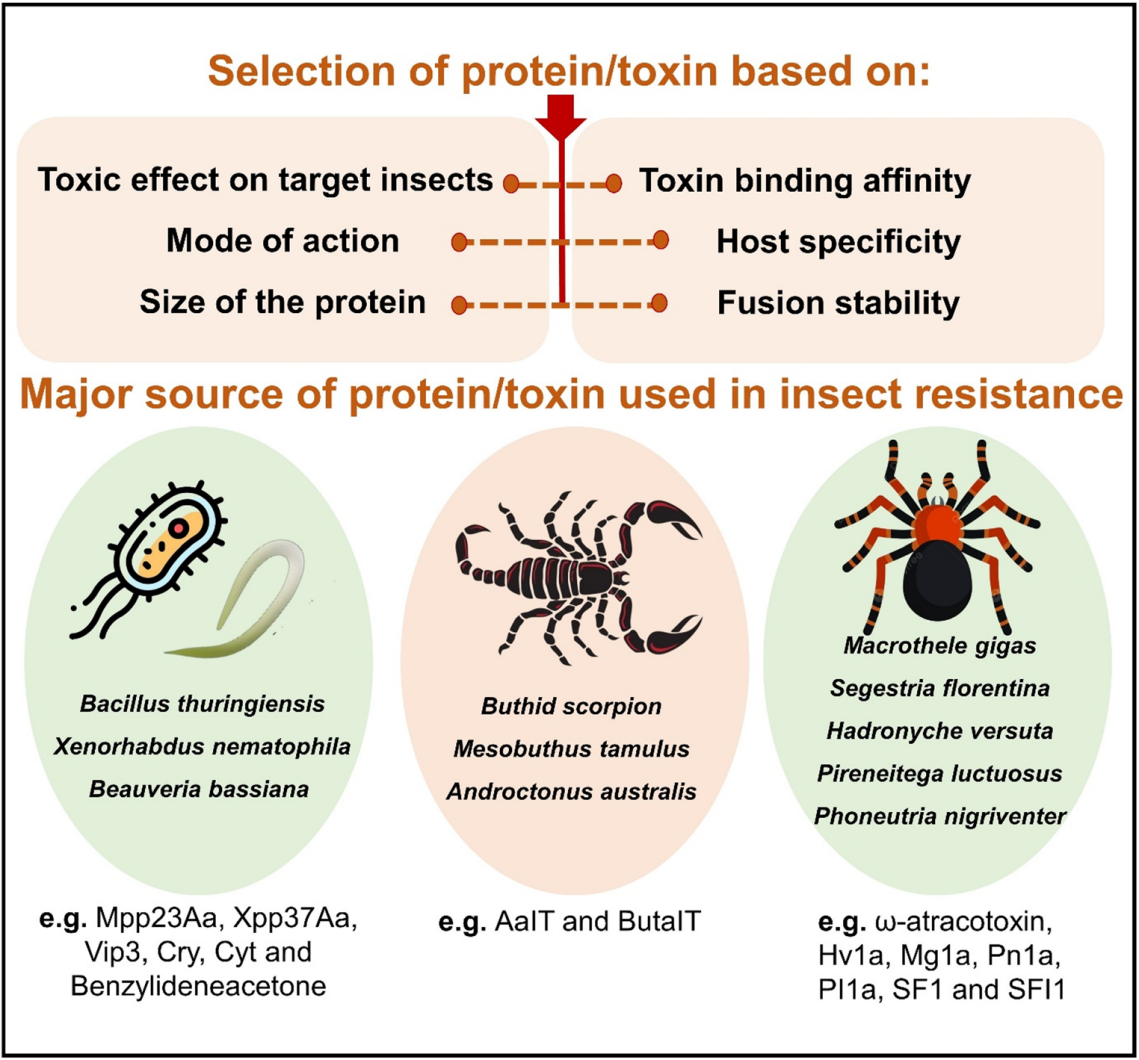
Selection criteria and major sources of insecticidal proteins/toxins. The top section outlines key criteria for selecting insecticidal proteins/toxins, such as toxicity, mode of action, protein size, binding affinity, host specificity, and fusion stability. The lower section presents major sources of these proteins/toxins: (1) microbes (e.g., *Bacillus thuringiensis*, *Xenorhabdus nematophila*, *Beauveria bassiana*); (2) scorpions (e.g., *Buthid scorpion*, *Mesobuthus tamulus*, *Androctonus australis*); and (3) spiders (e.g., *Macrothele gigas*, *Segestria florentina*, *Phoneutria nigriventer*), producing compounds like Cry, Vip3, AaIT, ω-atracotoxin, and others used in biopesticides and transgenic crops for insect resistance

When designing a fusion protein, multiple factors must be considered to ensure its effectiveness, stability, and specificity. Among these, the selection of constituent proteins or protein-coding genes is a critical step, largely determined by the source and intended biological function of the proteins (Fig. 2). Additionally, the size of the fusion protein and the overall vector should be sufficiently small to facilitate efficient handling and transformation. A well-optimized construct ensures efficient delivery into plant cells and facilitates successful insect control. Target pest specificity and toxin binding affinity are also crucial; the fused protein should precisely target specific insect pests while avoiding non-target and beneficial organisms. Various strategies, such as modifying receptor-binding domains, have been employed to enhance specificity and minimize off-target effects. Protein domains play a key role in generating functional fusion proteins, with toxic gene domains being widely used to improve host selectivity and binding affinity. For instance, the *Cry1Ac* gene contains three distinct domains, among which domains I and II are commonly used in fusion proteins due to their significant roles in membrane insertion, pore formation, and receptor recognition (Boddupally et al. 2018; Tajne et al. 2012). Domain I is composed of alpha-helices and plays a significant role in membrane insertion and pore formation (Bravo et al. 2013), whereas domain II is composed of beta-sheets and is involved in receptor recognition and binding to insect midgut protein (Bravo et al. 2011). Typically, *Cry1Ac*’s domain III will be deleted, and the remaining two domains will be connected to another protein from a different species that plays a key role in insecticidal activity (Tajne et al. 2012). This strategic domain-swapping or fusion approach allows researchers to engineer proteins that retain the core pore-forming function while acquiring new receptor-binding capabilities, thereby overcoming resistance or expanding the host range (Tajne et al. 2012; Liu et al. 2022).

Another critical factor is the mode of action of toxins, as Cry proteins require activation in the insect gut before they exert their toxic effects. The effectiveness of fusion proteins can be further enhanced by incorporating carrier proteins, which facilitate transportation into target cells, thereby increasing toxicity and target pest specificity. Additionally, the selection of fusion partners is critical, as combining insecticidal proteins such as *Cry1B* and *Cry1Ab* can enhance potency and broaden the target pest range. Structural integrity and stability must also be ensured, as improper folding or instability can compromise biological activity. Computational modeling and structural biology tools are instrumental in designing stable fusion constructs. Linker design is another essential aspect that directly impacts the functionality of the fusion protein. The choice between flexible linkers (e.g., glycine-rich sequences), which allow independent domain movement, and rigid linkers (e.g., proline-rich sequences), which maintain fixed domain orientation, is dictated by the intended mechanism. For example, in a fusion protein designed for nematode management, a flexible glycine-serine linker allowed two proteinase inhibitors to function independently, while a rigid alpha-helical linker created a dual-inhibitor with a synergistic effect (Urwin et al. 1998). This demonstrates how linker selection is a strategic tool to optimize activity.

The mode of action of toxins is also important to enhance target pest specificity. Cry proteins function by being solubilized in the target insect’s digestive tract, activated by host proteases, interacting with cadherin receptors, lysing midgut epithelial cells, and producing pores (Bravo et al. 2007). In addition to cadherin, several midgut receptor proteins have been identified as key components of Cry toxin action over the past two decades. These include aminopeptidase N (APN), alkaline phosphatase (ALP), and ATP-binding cassette (ABC) transporters, which mediate toxin binding, oligomerization, membrane insertion and pore formation in the insect midgut epithelium (Bravo et al. 2011; Pardo-López et al. 2013; Adang et al. 2014). Notably, mutations or altered expression of ABC transporters, particularly ABCC2 and ABCC3, have been strongly linked to field-evolved resistance to Cry toxins in several lepidopteran pests (Gahan et al. 2010). Collectively, these receptor-mediated interactions contribute to the high specificity and reduced off-target effects of Cry toxins. Additionally, carrier proteins have been demonstrated to enhance target pest specificity and toxicity (Fitches et al. 2002). Fusion of toxic proteins with suitable carrier proteins facilitates their efficient transportation of fusion proteins into target cells, resulting in increased target pest specificity and toxicity. When designing such fusion proteins, it is essential to ensure they are widely applicable. Several fusion proteins with broad spectrums have been created in rice (Gao et al. 2011; Quilis et al. 2014).

Optimizing the expression system is equally important, as baculovirus expression vectors in insect cells have proven effective for producing recombinant proteins with proper post-translational modifications. In plant-based fusion protein systems, promoter architecture plays a critical role in determining spatial and temporal expression patterns. Constitutive promoters such as *CaMV35S* enable broad expression but may increase metabolic burden and non-target exposure, whereas tissue-specific promoters (e.g., phloem- or green tissue-specific promoters) restrict expression to relevant feeding sites of target insects. Inducible promoters further allow conditional expression in response to biotic stress, reducing unintended ecological impacts. The choice and combination of regulatory elements therefore directly influence fusion protein efficacy, biosafety, and durability of insect resistance (Potenza et al. 2004; Venter 2007). Environmental risk assessment is necessary to evaluate potential non-target impacts, ensuring that fusion proteins do not negatively affect beneficial organisms or ecological balance. Furthermore, resistance management strategies should be integrated by designing fusion proteins with multiple insecticidal modes of action to reduce the likelihood of resistance development in insect populations. Lastly, broad-spectrum fusion proteins have been successfully developed in crops like rice, demonstrating their potential for widespread application in pest control. By incorporating these critical considerations, fusion protein design can be refined to enhance specificity, efficiency, and long-term sustainability in insect-resistant crop development. The major toxin components and their mechanisms involved in fusion protein engineering for insect resistance are summarized in Table 1.

**Table 1:** Toxin components and mechanisms for insect resistance in fusion protein engineering

Source	Toxin	Target insect	Mode of action	Reference
*Androctonus australis*	Neurotoxin (AaIT)	*Helicoverpa armigera*, *Bemisia tabaci* and *Nilaparvata lugens*	Blocking voltage-gated sodium channels	Liu et al. (2016b)
*Bacillus thuringiensis*	crystal (Cry) and cytolytic (Cyt)	Lepidopteran, Coleopteran and Dipteran pest	Lyse midgut epithelial cells	Bravo et al. (2007)
*Bacillus thuringiensis*	δ-endotoxin	*Spilarctia obliqua* and *Spodoptera exigua*	Target to epithelium cells of insect gut	Majumder et al. (2020)
*Bacillus thuringiensis*	Mpp23Aa and Xpp37Aa	*Cylas puncticollis* and *Anthonomus grandis*	Binds to brush border membrane vesicles	Hernández-Martínez et al. (2020); de Oliveira et al. (2023)
*Bacillus thuringiensis*	Vegetative insecticidal protein3 (Vip3)	Lepidopteran pests	Feeding inhibition, gut paralysis	Gupta et al. (2021)
*Beauvaria bassiana*	Huwentoxin-I	Lepidoptera	Block high-voltage activated calcium channels	Xia et al. (2009)
*Buthid scorpion*	*Androctonus australis* insect toxin (AaIT)	Chewing and sucking pests	Highly specific for the sodium channel	Liu et al. (2016b)
*Hadronyche versuta*	Neurotoxin (ω-hexatoxin-Hv1a)	*Aethina tumida*	Blocking voltage-gated calcium channels	Powell et al. (2020)
*Hadronyche versuta*	Neurotoxins (ω-ACTX-Hv1a)	*Myzus persicae*	Calcium channel blocker in the insect central nervous system	Nakasu et al. (2014)
*Hadronyche versuta*	ω-atracotoxin	*Phenacoccus solenopsis*	-	Javaid et al. (2018)
*Macrothele gigas*	µ-hexatoxin-Mg1a	-	Blocking voltage-gated sodium channels	Corzo et al. (2003)
*Mesobuthus tamulus*	Neurotoxins (ButaIT)	*Lacanobia oleracea*	Target-specific modulation of ion channel function	Trung et al. (2006)
*Monocotyledonous plants*	*Galanthus nivalis* agglutinin (GNA)	Cotton aphids and *Plutella xylostella*	Bind to chitin in the peritrophic matrix	He et al. (2022)
*Phoneutria nigriventer*	δ-ctenitoxin-Pn1a	-	Blocking voltage-gated sodium channels	Figueiredo et al. (1995)
*Pireneitega luctuosus*	δ-amaurobitoxin-PI1a	*Mamestra brassicae*	Target insect sodium channels	Yang et al. (2014)
*Pireneitega luctuosus*	δ-amaurobitoxin-PI1a or ω-hexatoxin-Hv1a	*Myzus persicae*	Target sodium and calcium ion channels	Yang et al. (2015)
*Pireneitega luctuosus*	ω-hexatoxin-Hv2a	-	Blocking voltage-gated calcium channels	Wang et al. (2001)
*Pireneitega luctuosus*	ω-theraphotoxin-Hs2a	-	Voltage-gated calcium channels	Deng et al. (2008)
*Segestria florentina*	Neurotoxin (SF1)	*Myzus persicae* and *Nilaparvata lugens*	Voltage-dependent calcium channels	Down et al. (2006)
*Segestria florentina*	Neurotoxin (SFI1)	*Lacanobia oleracea*	Potential to act as a carrier to deliver fused peptides to the circulatory system of target insect species	Fitches et al. (2004)
*Xenorhabdus nematophila*	Benzylideneacetone	*Plutella xylostella*	Inhibit PLA2 and prevent eicosanoid biosynthesis	Kim and Kim (2011)

## Application of fusion proteins in insect pest management

Fusion proteins can represent a green alternative to existing management strategies. Due to their specificity and effective target mechanism, fusion proteins can reduce pests without harming their natural enemies. For instance, the insecticidal fusion protein *Hv1a/GNA* exerted minimal impact on the parasitoid *Eulophus pennicornis*, a natural enemy of the pest *Lacanobia oleracea*, highlighting its potential as a biocontrol agent (Nakasu et al. 2016). The application of fusion proteins in insect pest resistance offers an environmentally friendly approach to pest management, minimizing reliance on harmful chemical pesticides, and promoting the sustainability of agroecosystems.

### Fusion protein crops based on Bt

*B. thuringiensis* (Bt) is a soil-dwelling, Gram-positive bacteria that produces unique crystalline proteins. These proteins possess remarkable insecticidal properties, exhibiting severe toxicity towards certain classes of insect pests (Panwar et al. 2018). In genetically engineered crops that are resistant to insects, the insecticidal activity is derived from specific genes within the Bt bacteria. These genes encode crystalline protoxin proteins that form parasporal crystals, which are responsible for conferring the pest-resistant trait in such crops (Palma et al. 2014). While the introduction of insecticidal cry genes into crop plants has proven to be an effective strategy for combating various insect pests, certain agricultural pests have demonstrated an ability to adapt and develop resistance to these toxins. Field-evolved resistance to Cry toxins has been reported in several major agricultural pests, including resistance to Cry1Ac in *H. armigera* associated with Bt cotton cultivation, resistance to Cry1F and Cry1Ab in *S. frugiperda* and resistance to Cry3Bb1 in the western corn rootworm *(Diabrotica virgifera virgifera*). These documented field cases demonstrate the limitations of single Cry gene deployment and highlight the need for improved resistance-management strategies such as fusion proteins and pyramided constructs (Tabashnik et al. 2013; Gassmann et al. 2011; Carrière et al. 2015). As a result, these resilient pests can overcome the protective measures implemented through cry gene technologies and potentially cause significant damage to crop yields.

Transgenic rice lines expressing a fusion protein of two different Bt toxins, *Cry1Ac* and *Cry1I*-like protein using a green tissue promoter, were highly effective against the rice leaf folder and the striped stem borer in both laboratory and field bioassays (Yang et al. 2014). Notably, a chimeric protein, Cry1Ab-1Gc, was engineered by replacing domain III of Cry1Ab with that of Cry1Gc. This fusion protein exhibited high toxicity against lepidopteran pests, and transgenic rice expressing Cry1Ab-1Gc demonstrated significant resistance to rice stem borers. Similarly, transgenic maize expressing this fusion protein showed enhanced resistance to the Asian corn borer, indicating its potential for broader application in crop protection (Liu et al. 2022).A fusion gene SP-Cry1Ca-CBM11, consisting of the Bt gene *Cry1Ca* linked to a secretion signal peptide and a carbohydrate-binding module 11 in homozygous transgenic rice lines, exhibited high resistance against major rice lepidopteran pests. The *Cry1Ca* content varied among different plant tissues, with the highest levels observed in leaves (up to 17.55 µg/g in transformant 21H037). The fusion Bt protein successfully accumulated on cell walls and imparted high resistance against lepidopteran pests without affecting agronomic traits (Li et al. 2024).

A fusion gene comprising the insecticidal *Cry1Ac* protein from *B. thuringiensis* (Bt) and the carbohydrate-binding domain of garlic lectin (*ASAL*) in transgenic rice lines successfully conferred enhanced resistance against three major rice pests: yellow stem borer (YSB), leaf folder (LF), and brown planthopper (BPH). Honeydew assays revealed a significant decrease in the feeding ability of BPH on transgenic plants. Ligand blot analysis using BPH insects feeding on transgenic rice plants showed that the fusion protein could also bind to additional receptors in insects (Boddupally et al. 2018). Transgenic rice plants expressing a fusion protein comprising the insecticidal proteins *Cry1Ab* and *Vip3A* exhibited high resistance against two major rice pests, the Asiatic rice borer (*Chilo suppressalis*) and the rice leaf folder (*Cnaphalocrocis medinalis*) (Xu et al. 2018). Bt-based fusion protein strategies, including Cry–Cry and Cry–Vip combinations, have been reported to enhance insecticidal efficacy and improve resistance durability compared with single-gene Bt crops (Bravo et al. 2011; Sainsbury et al. 2012; Liu et al. 2016a; Javaid et al. 2018; Sukiran et al. 2023).

### Lectin as a carrier for fusion proteins

The use of lectins as carrier proteins is a direct application of the strategy to enhance oral bioavailability and systemic delivery as described in Sect. 1.1. Lectins are a unique group of proteins and glycoproteins with potent biological activity. They are found in several crops like snowdrop (*Galanthus nivalis* L.), wheat (*Triticum aestivum* L.) and many legumes. *Galanthus nivalis* agglutinin (GNA), a small 14-kDa mannose-specific lectin from the snowdrop plant, is often used as a carrier for fusion protein delivery in insect pests. An engineered fusion protein containing snowdrop lectin (GNA) as a carrier to deliver an insect growth-regulating neuropeptide (Manse-AS) into *Lacanobia oleracea* larvae showed promising results by significantly affecting its feeding and growth in the 5th instar (Fitches et al. 2002). Additionally, transplastomic approaches have been employed to express fusion proteins in plant chloroplasts, leading to high-level accumulation of insecticidal proteins and enhanced pest resistance. For instance, plastid-expressed double-stranded RNAs targeting essential genes in the Colorado potato beetle conferred effective protection, with transplastomic potato plants exhibiting an 83% larval mortality rate (Zhang et al. 2015). Nevertheless, the specificity of lectins may limit their effectiveness against a broad spectrum of pests, and there is a risk of insects developing resistance over time. These limitations highlight the need for careful design and deployment of lectin-based fusion proteins, which have nevertheless been shown to enhance delivery efficiency and insecticidal performance by facilitating toxin uptake in insect gut tissues (Powell et al. 2020; Liu et al. 2016a; Javaid et al. 2018).

### Scorpion toxin-based fusion proteins

Scorpion neurotoxins are potent but typically ineffective upon ingestion. Their fusion with carrier proteins like GNA exemplifies the oral bioavailability strategy, enabling systemic delivery to the insect nervous system (Ortiz and Possani 2015). Scorpion venoms are remarkably diverse, containing a plethora of polypeptides that exhibit an array of biological activities. Among these polypeptides are neurotoxins that selectively target and modulate the function of specific ion channels (Escoubas et al. 2000; Rash and Hodgson 2002). Despite the similarities in ion channels between invertebrates and vertebrates, many of these scorpion neurotoxins demonstrate a remarkable specificity towards species or groups of species. This selective action of scorpion toxins, coupled with the properties of similar proteins, holds significant promise for the development of safer alternatives to broad-spectrum insecticides, which often lack such specificity (Trung et al. 2006). Recombinant fusion proteins, formed by combining insecticidal toxins from venomous scorpions with carrier proteins like GNA, have shown promising results in conferring insect resistance to transgenic plants. The ButaIT/GNA fusion protein, comprising the ButaIT toxin from the red scorpion (*Mesobuthus tamulus*) and GNA, exhibited acute toxicity when injected into tomato moth (*L. oleracea*) larvae, causing paralysis, mortality, and stunted growth (Trung et al. 2006). Additionally, oral administration of ButaIT/GNA led to chronic toxicity, decreasing survival and weight gain, with the fusion protein being detected in the larvae’s hemolymph, indicating successful transport across the gut barrier.

Transgenic tobacco, Arabidopsis, and rice plants were developed expressing the AaIT/GNA fusion protein, which combines the insecticidal scorpion venom neurotoxin (*Androctonus australis* toxin, AaIT) with GNA (Liu et al. 2016b). These transgenic plants exhibited increased resistance and toxicity against the chewing pest cotton bollworm (*H. armigera*) compared to plants expressing AaIT or GNA alone. Additionally, transgenic tobacco and rice plants expressing AaIT/GNA showed enhanced resistance and toxicity against the sucking pests whitefly (*Bemisia tabaci*) and rice brown planthopper (*N. lugens*), respectively. The insecticidal activity of the ButaIT/GNA fusion protein from the red scorpion *M. tamulus* and GNA was compared with SFI1/GNA, a fusion comprising a venom toxin (SFI1) from the European spider *Segestria florentina* and GNA (Fitches et al. 2010). Injection bioassays revealed that both ButaIT/GNA and SFI1/GNA were toxic against larvae of Lepidoptera, adults of Diptera and Coleoptera, larvae of Coleoptera, and nymphs of Dictyoptera. Notably, ButaIT/GNA exhibited higher toxicity than the previously studied SFI1/GNA fusion protein across all tested insects. The *BjαIT* gene, encoding a scorpion long-chain insect neurotoxin, was cloned into the pET32 expression vector and expressed as a thioredoxin (Trx)-BjαIT (rBjαIT) fusion protein in *Escherichia coli* (Li and Xia 2018). Their findings revealed that injecting the rBjαIT fusion protein induced obvious neurotoxic symptoms and led to the death of locust (*Locusta migratoria*) larvae and any dietary toxicity was not observed in locusts when the fusion protein was ingested. An active form of rBjαIT can be efficiently obtained from an *E. coli* expression system, which could be beneficial for evaluating its insecticidal potential against agricultural insect pests. Recent studies have continued to explore the potential of scorpion toxin-based fusion proteins to enhance insect resistance in crop plants. For instance, a scorpion toxin LqhIT2 has been expressed in genetically modified systems such as baculoviruses, demonstrating enhanced insecticidal activity that leads to increased larval mortality in target pests (van Beek et al. 2003) and similar improvements in efficacy and specificity have been reported for other scorpion toxin–based fusion proteins across different delivery platforms (Wang et al. 2001; Corzo et al. 2003).

### Spider venom-based fusion proteins

Like scorpion toxins, spider venom peptides are potent neurotoxins that require a delivery system. Fusion with lectins like GNA facilitates oral delivery, and the diversity of spider toxins allows for the targeting of a broad spectrum of insect ion channels (Nakasu et al. 2014). Spider venom is a complex mixture of various toxins that can inflict severe neurological and biochemical effects on animals, including mammals (Beleboni et al. 2004). This venom contains a diverse array of toxic polypeptides that can induce paralysis in the prey. Fusion proteins combining insecticidal toxins from venomous spiders with carrier proteins like GNA have demonstrated enhanced oral toxicity against various insect pests. The PI1a/GNA fusion, comprising the spider venom peptide delta-amaurobitoxin-PI1a (PI1a) targeting insect voltage-gated sodium channels and GNA, caused 100% mortality in 3rd instar cabbage moth (*Mamestra brassicae*) larvae within 6 days and reduced survival, growth, and feeding in later instars. Interestingly, PI1a/GNA exhibited oral toxicity against lepidopteran, dipteran (*Musca domestica*; housefly), and hemipteran (*Acyrthosiphon pisum*; pea aphid) insects (Yang et al. 2014). Researchers also studied the insecticidal activity of PI1a/GNA and Hv1a/GNA, containing the calcium channel-targeting ω-hexatoxin-Hv1a (Hv1a), against the peach-potato aphid (*Myzus persicae*), including insecticide-resistant strains (Yang et al. 2014). While mutations in the sodium channel conferring pyrethroid resistance led to cross-resistance against PI1a/GNA, these mutations did not affect the toxicity of Hv1a/GNA. The SFI1/GNA fusion protein, comprising the *Segestria florentina* toxin 1 (SFI1) neurotoxin and GNA, caused significant mortality in the rice brown planthopper (*N. lugens*) and reduced survival of the peach-potato aphid (*M. persicae*) when incorporated into artificial diets (Down et al. 2006). Similarly, when injecting Hv1a/GNA fusion protein (spider-venom peptide ω-hexatoxin-Hv1a (Hv1a) and GNA) into *Mamestra brassicae* (cabbage moth) larvae, it exhibited similar insecticidal activity as recombinant Hv1a alone (Fitches et al. 2012). However, when delivered orally via droplet feeding or leaf disc assays, Hv1a/GNA, but not Hv1a alone, caused significant reductions in the growth and survival of *M. brassicae* larvae. Studies have also demonstrated the potential of using GNA as a carrier to deliver the insecticidal spider venom neurotoxin SFI1 to the hemolymph of lepidopteran larvae (Fitches et al. 2004). While neither GNA nor SFI1 alone showed acute toxicity when fed to tomato moth (*Lacanobia oleracea*) larvae, feeding the SFI1/GNA fusion protein at 2.5% of dietary proteins imparted 100% mortality in first stadium larvae within 6 days. The fusion of the spider venom peptide GS-ω/κ-HxTx-Hv1h (HxTx-Hv1h), the active ingredient in the insecticidal product Spear^®^-T, with GNA exhibited significantly higher oral toxicity against pea aphid (*Acyrthosiphon pisum*) and peach-potato aphid (*M. persicae*) compared with venom peptide alone (Sukiran et al. 2023). Similar enhancements in delivery efficiency and insecticidal efficacy have also been reported for other spider venom- based fusion proteins (Liu et al. 2016a).

### TAT-PTD–diapause hormone based fusion protein

Protein transduction domains (PTDs) have been explored as carriers to enhance the delivery and bioavailability of bioactive peptides and proteins across biological membranes. For instance, the TAT-PTD from the human immunodeficiency virus-1 transactivator of transcription can potentially improve the oral delivery of diapause hormone (caDH) from *Clostera anastomosis* to the cotton bollworm (Zhou et al. 2015). An oral administration of TAT-caDH fusion protein resulted in larval growth inhibition in *H. armigera*, with delayed larval duration and decreased pupation rates observed under both development-promoting and diapause-inducing conditions. Interestingly, while no significant difference in diapause rate was observed between TAT-caDH-treated and control pupae maintained under diapause-inducing conditions, the study demonstrated the effectiveness of orally delivered TAT-caDH in affecting larval development.

Insect kinins, owing to their diuretic activity, suppress weight gain, and function as antifeedants in insects. To explore the potential of the TAT-fusion approach for the oral delivery of diuretic peptides to pest insects, researchers selected the HezK I peptide from *Helicoverpa zea* as a representative of the kinin family (Zhou et al. 2017). A fusion gene, TAT-HezK I, was constructed and introduced into tobacco plants. Additionally, to enhance the stability of TAT-HezK I, another fusion protein incorporating HezK I, transactivator of transcription (TAT), and cowpea trypsin inhibitor (CpTI) was developed. The toxicity of different transgenic tobacco lines was evaluated against *H. armigera*, revealing that the transgenic expression of TAT-HezK I effectively conferred toxicity to insects through larval feeding. Furthermore, the fusion of TAT-HezK I and CpTI exhibited greater toxicity than CpTI alone, improving both stability and bioavailability upon oral administration. TAT-PTD–diapause hormone–based fusion proteins have been shown to enhance cellular uptake and biological activity of peptide hormones in transgenic plants, resulting in increased tolerance to herbivorous insect attack (Deng et al. 2008; Xia et al. 2009). These studies demonstrate that cell-penetrating peptide–mediated delivery can be effectively exploited to improve the performance of peptide-based fusion proteins for insect resistance, with representative fusion constructs, transformation systems and target pests summarized in Table 2.

**Table 2:** Fusion protein applications for insect resistance in field crops including transformation systems and target pests

Crop	Fusion protein	Vector	Promoter	Terminator	Resistance to Insects	References
Arabidopsis	ω-ACTX-Hv1a and GNA	-	CaMV 35S	-	*Myzus persicae*	Nakasu et al. (2014)
Jute	Cry1Ab and Cry1Ac	-	CaMV 35S	NOS	*Spilarctia obliqua* and *Spodoptera exigua*	Majumder et al. (2020)
Maize	cry1Ac and hwtx-I	pXL43	Native	35S	-	Xia et al. (2009)
Maize	cry1B and cry1Ab	-	Ubi	NOS	*Diatraea grandiosella*, *Diatraea saccharalis* and *Spodoptera frugiperda*	Bohorova et al. (2001)
Maize	Cry1Ab and Cry1Gc	pTF101.1	Ubi	-	*Scirpophaga incertulas*	Boddupally et al. (2018)
Maize	cry2Ab, vip3A and cp4epsps	-	Ubi, Actin2 and CaMV 35S	CaMV 35S poly A, Hsp17 and NOS	*Mythimna separata*, *Helicoverpa armigera* and *Spodoptera frugiperda*	Liu et al. (2023)
Pulses	PAF/GNA	pGAPZαB	-	-	*Acyrthosiphon pisum* and *Myzus persicae*	De-Thier et al. (2023)
Rice	AaIT/GNA	p1301	Ubi	NOS	*Helicoverpa armigera*, *Bemisia tabaci*	Liu et al. (2016b)
Rice	Bt Cry1Ac and ASAL	-	CaMV 35S	polyA	*Scirpophaga incertulas*, *Cnaphalocrosis medinalis* and *Nilaparvata lugens*	Boddupally et al. (2018)
Rice	Cry1Ab and Cry1Gc	pTF101.1	Ubi	-	*Scirpophaga incertulas*	Liu et al. (2022)
Rice	CrylAb and Cry9Aa	-	Polyubi-1	PEPC	*Scirpophaga incertulas*, *Cnaphalocrosis medinalis*	Gao et al. (2011)
Rice	Cry1Ab and Vip3A	-	PActin1	PEPC	*Chilo suppressalis* and *Cnaphalocrosis medinalis*	Xu et al. (2018)
Rice	Cry1Ab and Vip3H	-	Ubi	-	*Chilo suppressalis* and *Sesamia inferens*	Chen et al. (2010)
Rice	MPI and PCI	-	MPI promoter	MPI	*Chilo suppressalis*	Quilis et al. (2014)
Tobacco	cry1Ac and hwtx-I	-	35S	NOS	-	Xia et al. (2009)
Tobacco	ω-atracotoxin and onion leaf lectin	-	CaMV 35S	NOS	*Phenacoccus solenopsis*	Javaid et al. (2018)
Tomato	cry1Ac and hwtx-I	-	35S	NOS	-	Xia et al. (2009)
Tomato	crylC-crylA(b)	-	35S	NOS	*Spodoptera exigua*,* Heliothis virescens* and *Manduca sexta*	Van Der Salm et al. (1994)

Cauliflower mosaic virus (*CaMV*), Maize Proteinase Inhibitor (*MPI*), Nopaline synthase (*NOS*), Phosphoenolpyruvate carboxylase (*PEPC*), and Ubiquitin (*Ubi*)

## Fusion proteins against other crop production challenges

Beyond combating insect pests, fusion proteins hold immense potential in addressing various other agricultural challenges including diseases, nematodes, weeds and abiotic stresses. These engineered proteins can be tailored to enhance crop tolerance to abiotic stresses like drought, salinity, extreme temperatures and heavy metals. Additionally, they offer opportunities for improving nutrient uptake efficiency, increasing biomass yield, and even conferring resistance against diseases. Harnessing the versatility of fusion proteins paves the way for innovative solutions in sustainable agriculture.

### Fusion proteins and disease management

Plant pathogens, particularly fungi and viruses, are becoming increasingly widespread and severe, posing a significant threat to agriculture due to the lack of effective agrochemicals and the absence of resistance or immunity in host plants. One potential solution to combat these plant diseases is the creation of fusion or chimeric proteins (Ingham and Moore 2007).

These proteins are formed by combining two protein sequences with similar or complementary functions, resulting in enhanced efficacy against pathogenic infections. By harnessing the synergistic effects of these chimeric proteins, scientists aim to develop innovative strategies to protect crops and mitigate the impact of plant pathogens on agricultural productivity.

In rice, the expression of the mpi-pci fusion gene confers enhanced resistance to the fungal pathogen *Magnaporthe oryzae*, the causal agent of rice blast disease (Quilis et al. 2014). Additionally, the OsWAK1-GFP fusion protein localizes to the cell surface and is associated with the cell wall; transgenic rice lines overexpressing it showed enhanced resistance to rice blast (Li et al. 2009). In wheat, transgenic plants expressing a fusion protein consisting of a Fusarium-specific recombinant antibody from chicken and an antifungal peptide from *Aspergillus giganteus* exhibited enhanced resistance against Fusarium head blight caused by *Fusarium asiaticum* (Li et al. 2008). Transgenic tobacco plants expressing a fusion protein of two plant defensins, Tfgd2 and RsAFP2, demonstrated improved protection against fungal pathogens *Rhizoctonia solani* and *Phytophthora parasitica*, as well as resistance to the herbivore *Spodoptera litura* (Vasavirama and Kirti 2013). Furthermore, the introduction of a tobacco phylloplanin-GFP fusion gene targeted to the apoplast enhanced resistance to blue mold disease caused by *Peronospora tabacina* (Kroumova et al. 2013).

In cruciferous crops, the antimicrobial protein PBT1 from *Bacillus subtilis* strain XF-1, when introduced into the biocontrol agent *Pseudomonas fluorescens* 2P24, enhanced its antagonistic activity against the clubroot pathogen *Plasmodiophora brassicae* (Zhao et al. 2016). The Hcm1 fusion protein, created by fusing the Hpa1 protein from *Xanthomonas oryzae* pv. *oryzae* with α-helices of cecropin A and melittin, demonstrated effective resistance against tobacco mosaic virus in tobacco, *Magnaporthe oryzae* in rice, and *Ralstonia solanacearum* in tomato (*Solanum lycopersicum* L.) (Che et al. 2011). The super-Blad chimeric protein (fusion of Blad with SP10-5) exhibited antibacterial effects against *Xanthomonas* spp. and *Pseudomonas syringae*, as well as the ability to suppress the proliferation of yeast and filamentous fungi (Pinheiro et al. 2018). The TDEF1 fusion protein, derived from *Trichosanthes kirilowii* defensin, exhibited antifungal activity against the plant pathogen *Fusarium oxysporum* (Da-Hui et al. 2007). Andersen et al. (2020) provide insights into the evolutionary arms race between plants and pathogens, revealing extensive gene clustering and the emergence of NLR-ID fusion proteins in wheat, suggesting functional diversification of the NLR immune receptors to combat pathogens.

### Nematode management using fusion proteins

The co-delivery of multiple proteinase inhibitors has been explored as a strategy to enhance the efficacy of plant defense against pathogens and pests. Researchers have studied the co-delivery of two proteinase inhibitors, a cysteine inhibitor (Oc-IDD86) and a serine inhibitor (CpTI), in *Arabidopsis* via translational fusions using different peptide linkers (Urwin et al. 1998). The PsMTa (plant metallothionein-like protein) linker yielded single inhibitors, while the GO (Galactose oxidase) linker produced a dual inhibitor. Uptake of inhibitors by nematodes occurred with the GO linker but not PsMTa. Interestingly, the CpTI from PsMTa affected nematode development externally. The dual inhibitor linked by GO showed an additive effect, highlighting the potential of co-delivering multiple effectors against plant pathogens. A summary of fusion proteins employed for disease and nematode resistance in field crops, along with their transformation constructs and target organisms, is presented in Table 3.

**Table 3:** Fusion proteins utilized in field crops for disease and nematode resistance with their transformation construct information

Crop	Fusion protein	Vector	Promoter	Terminator	Resistance to pathogen	Reference
Arabidopsis	Oc-IDD86 and aCpTI	-	-	-	Cyst nematodes and root-knot nematodes	Urwin et al. (1998)
Black gram	Bacterial chitinase (*ChiB*) gene	-	35S	-	Powdery mildew *Erysiphae polygoni*	Das (2018)
Wheat	NLR-ID	pCRTM8/GW/TOPO vector	-	-	No specific pathogen mentioned	Andersen et al. (2020)
Crucifer’s family	Pbt1	pQE81-L and pRK415	-	-	*Plasmodiophora brassicae*	Zhao et al. (2016)
Rice	Hcm1	pET30a	-	-	*Magnaporthe oryzae*	Che et al. (2011)
Rice	mpi-pci	MPI-2 A-PCI	MPI	MPI	*Magnaporthe oryzae* (Rice Blast)	Quilis et al. (2014)
Rice	OsWAK1 fused with GFP	pCAMBIA1305-1	CaMV 35S	-	*Magnaporthe oryzae* (Rice Blast)	Li et al. (2009)
Tobacco	Hcm1	pET30a	-	-	Tobacco mosaic virus (TMV)	Da-Hui et al. (2007)
Tobacco	T-phylloplanin-GFP	pKM24KH	MMV	-	*Peronospora tabacina* (blue mold disease)	Kroumova et al. (2013)
Tobacco	ORF13 and RSL1	-	-	-	*Sclerotinia sclerotiorum*, *Rhizoctonia solani* & *Pythium* sp.	Biliarski et al. (2020)
Tobacco	Tfgd2-RsAFP2	pRD-400	CaMV 35S	CaMV 35S poly A signal	*Rhizoctonia solani* and *Phytophthora parasitica* var. nicotianae	Vasavirama and Kirti (2013)
Tomato	Hcm1	pET30a(+)	-	-	*Ralstonia solanacearum*	Che et al. (2011)
Wheat	AG-scFv	pAHC25	Ubi	NOS	*Fusarium asiaticum*	Li et al. (2008)
Wheat	AFP-scFv	pET22b and pTRAkc	-	-	*F. graminearum* and *F. oxysporium* f.sp. matthiolae	Peschen et al. (2004)

Cauliflower mosaic virus (CaMV), Modified Mirabilis mosaic virus (*MMV*), Maize Proteinase Inhibitor (*MPI*), Nopaline synthase (*NOS*), and Ubiquitin (*Ubi*)

### Use of fusion proteins in abiotic stress management

Fusion proteins are not only effective in combating biotic stress but also hold significant potential in addressing other agricultural challenges. One such application is enhancing protein content in crops. Overexpression of the synthetic fusion genes *TKTKK1* and *TKTKK2*, encoding proteins with high lysine/threonine content, in transgenic rice seeds led to substantial increases in lysine, threonine, total amino acids, and crude protein compared to wild-type controls (Jiang et al. 2016).

Fusion proteins have also been explored for improving heavy metal tolerance in plants. The SALI3-2-GFP fusion protein, derived from the soybean BURP-domain protein SALI3-2, conferred tolerance to cadmium and copper stresses in *Arabidopsis thaliana* by facilitating heavy metal sequestration in roots, thereby reducing their transport to aerial tissues (Tang et al. 2014). A fusion protein approach was utilized to elucidate the subcellular localization and function of CjNRAMP1-GFP, a metal transporter from the metal-accumulating legume *Crotalaria juncea* (Nakanishi-Masuno et al. 2018). The fusion protein localized to the plasma membranes of plant cells, and complementation studies confirmed its ability to transport Fe and Cd, leading to increased metal accumulation and enhanced Cd tolerance.

Fusion proteins have also been employed to study and improve abiotic stress tolerance in plants. For instance, the OsNAC5-GFP fusion protein was used to investigate the subcellular localization and transcriptional activation activity of OsNAC5, a stress-responsive transcription factor in rice (Takasaki et al. 2010). Overexpression of OsNAC5 in transgenic rice enhanced tolerance to high salinity stress without affecting growth, highlighting the utility of the fusion protein strategy in improving abiotic stress tolerance. Fusion protein applications extending beyond insect resistance, including disease suppression, nematode management, and abiotic stress tolerance, have been documented across diverse crop systems (Bohorova et al. 2001; Figueiredo et al. 1995; Deng et al. 2008; Xia et al. 2009; Javaid et al. 2018), with representative examples summarized in Table 4.

**Table 4:** Fusion proteins utilized in other agricultural challenges

Crop	Fusion protein	Vector	Promoter	Role of Fusion protein	Reference
Arabidopsis	SALI3-2 fused with GFP	pET28a	T7	Facilitates heavy metal sequestration	Tang et al. (2014)
*Crotalaria juncea*	GFP-CjNRAMP1	-	-	Enhanced Cd tolerance	Nakanishi-Masuno et al. (2018)
Rice	OsNAC5-GFP, OsNAC6-GFP	pGHsGFP, pGHUsGFP, pBI35S-VP16AD, pGHU, pBCKH, pGBKT7, pBI221	OsNAC5, Ubiquitin CaMV 35S, OsNAC3, OsNAC4, and SNAC1	Tolerance to high salinity	Takasaki et al. (2010)
Rice	TKTKK1 and TKTKK2	-	35S	Increase lysine, threonine, and protein content	Jiang et al. (2016)

Cauliflower mosaic virus (*CaMV*), Cadmium (*Cd*), Green fluorescent protein (*GFP*)

## Challenges, advantages, and future perspectives of fusion protein technology

### Durability, safety and regulatory challenges

The long-term success of fusion protein technology hinges on its ability to overcome critical post-engineering and regulatory hurdles. A primary concern is durability, which is addressed by leveraging the inherent advantage of fusion proteins as a pyramiding strategy (Sainsbury et al. 2012). By combining proteins with distinct insecticidal modes of action, such as an ion-channel disrupter (e.g., Cry toxin) and a receptor blocker the technology creates a high genetic barrier, significantly delaying the evolution of pest resistance in the field (Wang et al. 2025). Simultaneously, rigorous safety assessments are paramount for market acceptance. Regulatory approval requires comprehensive testing to evaluate potential toxicity and allergenicity to mammals, alongside extensive analysis of impacts on non-target organisms, including vital pollinators and beneficial predatory insects (Cappa et al. 2022). In addition to these general safety assessments, fusion protein–based strategies may pose potential off-target risks due to altered binding specificity or enhanced stability of fused constructs, underscoring the need for systematic non-target risk assessment under laboratory and field conditions (Romeis et al. 2008; Devos et al. 2013). This necessitates scrutiny to ensure the targeted specificity of the fusion construct is maintained *in planta*. Finally, regulation presents a complex challenge, as bifunctional proteins must be assessed not just as two existing components but as a single, integrated entity (Sainsbury et al. 2012). Harmonizing the diverse global regulatory frameworks for genetically engineered crops, coupled with demonstrating stable and consistent expression of the fusion protein throughout the growing season, represents the final, crucial step toward the successful and sustainable deployment of this powerful biotechnological tool.

### Major advantages and limitations in the application of fusion proteins

**Fig. 3:**
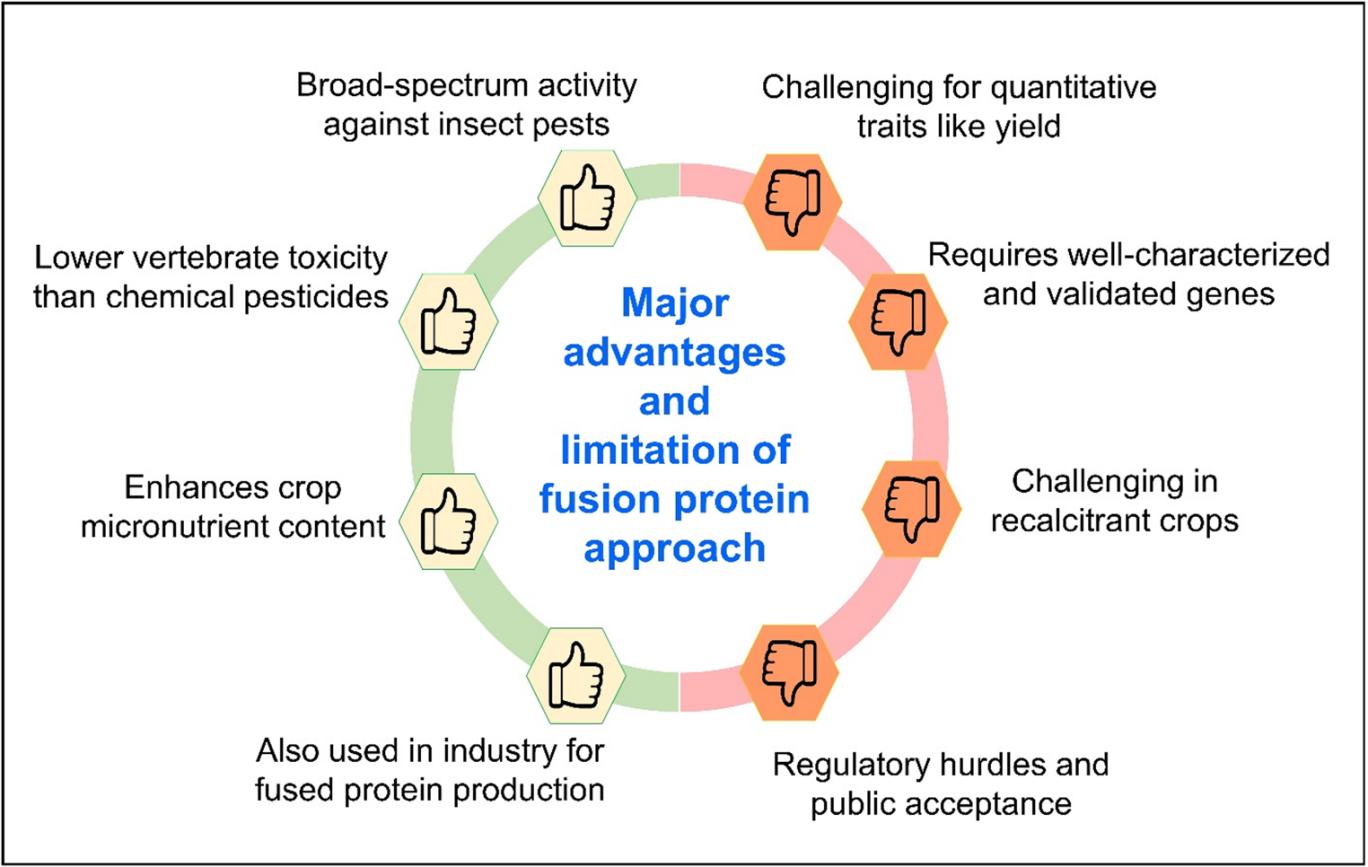
Major Advantages and Limitations of Fusion Protein Approach in Agriculture

The fusion protein approach in agricultural biotechnology offers several significant advantages (Fig. 3). Beyond agriculture, the fusion protein approach also finds applications in industry, enabling the production of valuable fused proteins for various biotechnological and industrial purposes, thereby expanding its economic potential. From an applied perspective, fusion protein technologies offer practical advantages in crop protection programs. These include improved overall efficacy through combined functional activities, reduced dependence on chemical insecticides, and enhanced durability of resistance when compared with single-gene approaches. The ability to tailor fusion constructs for specific crop–pest systems also provide flexibility in deployment, supporting more sustainable pest management strategies under diverse agricultural conditions. In addition, fusion protein strategies can enhance delivery efficiency, increase potency through synergistic interactions, incorporate multiple modes of action, and allow tissue-specific expression to improve safety by reducing non-target exposure.

Despite these advantages, the fusion protein approach faces several limitations and challenges. From a technical perspective, fusion protein performance may also be constrained by design-related factors such as linker complexity, improper protein folding, and susceptibility to degradation, which can affect stability and biological activity. One limitation is that incorporating fusion proteins may not directly address complex quantitative traits like crop yield, which are influenced by multiple genes and environmental factors, making it challenging to achieve significant yield improvements through this approach alone. Additionally, the fusion protein approach relies on the availability of well-understood and validated genes, limiting its application to traits or targets that are not yet fully characterized at the molecular level. Furthermore, some crop species are recalcitrant to genetic transformation techniques, making it difficult to introduce fusion protein construct into their genomes using current biotechnological methods.

The development and deployment of fusion protein technologies in new crops is particularly challenging due to significant limitations in specialized workforce availability, high development costs, and the extended duration required for research, regulatory approval, and commercialization processes. These resource constraints often restrict fusion protein applications to major commercial crops while neglecting orphan or regionally important crop species. Finally, the commercialization of crops produced through genetic engineering techniques, including fusion protein technology, faces regulatory hurdles and potential public skepticism or resistance due to concerns about biosafety and environmental impact. This necessitates effective regulatory frameworks and open communication with stakeholders to ensure responsible and sustainable adoption of this technology.

### Future directions and emerging opportunities

By fusing different protein domains with complementary functions, researchers can create engineered chimeric proteins with enhanced or synergistic activities. One approach is to combine stress-responsive transcription factors with functional proteins, such as enzymes involved in stress response pathways or proteins that provide structural protection. These fusion proteins could be designed to activate specific stress-responsive genes or pathways more effectively, leading to improved stress tolerance. Additionally, fusion proteins could be engineered to target specific cellular compartments or tissues, enhancing their efficacy and minimizing potential off-target effects. The advent of advanced biotechnological tools such as CRISPR/Cas9 genome editing has opened new opportunities for the precise integration of fusion protein constructs into crop genomes, enabling targeted insertion at defined loci to ensure stable expression, predictable inheritance, and reduced positional effects. CRISPR/Cas9 can be used to precisely insert or modify genes encoding fusion proteins, ensuring their stable expression and inheritance. A key challenge moving forward will be to develop efficient delivery systems without laborious transformation methods, streamlining the integration of fusion proteins into diverse crop varieties.

In addition to CRISPR/Cas9, emerging technologies like RNAi and gene drives offer exciting opportunities to complement fusion protein strategies. RNAi can be strategically integrated with fusion protein–based approaches by targeting complementary insect resistance or detoxification pathways, thereby enhancing the efficacy and durability of fusion protein-mediated pest control. Similarly, gene drives could be employed to propagate favourable traits, such as increased susceptibility to fusion protein-based toxins, across pest populations. These combined approaches can amplify pest control efficiency, reduce the likelihood of resistance development, and contribute to more robust pest management strategies. Furthermore, advancements in functional genomics enable rapid gene function characterization, accelerating the discovery and validation of new fusion protein components. Microbial sequencing initiatives continue to uncover previously uncharacterized toxin proteins with potential applications in fusion protein development. Integrated approaches that combine fusion protein strategies with other stress tolerance mechanisms, tolerance mechanisms, such as the modulation of phytohormone signalling, could lead to synergistic effects and more robust stress tolerance. Multidisciplinary collaborations involving molecular biologists, protein engineers, plant breeders, and agronomists will be crucial in translating the potential of fusion proteins into practical applications. Advances in artificial intelligence and computational biology provide valuable tools for the rational design of fusion proteins. Machine learning–based structure–function prediction, molecular docking, and in silico screening can guide domain selection, linker optimization, and binding specificity before experimental validation, thereby accelerating development and reducing trial-and-error.

## Conclusion

The development and application of fusion proteins in agriculture represent a promising biotechnological approach for sustainable insect pest management. Rather than introducing a new technology, this review consolidates existing evidence to highlight how fusion protein engineering has matured into a practical biotechnological tool for crop protection, bridging fundamental research and field-level applications. By combining the functional properties of multiple proteins, these engineered molecules offer a versatile and effective solution to target specific insect pests while minimizing environmental impact. Numerous fusion proteins have already been successfully developed and deployed in various crop plants, demonstrating their broad-spectrum activity against major lepidopteran, coleopteran, and hemipteran pests. Beyond insect pest management, fusion proteins hold immense potential for addressing other agricultural challenges, such as enhancing disease resistance, nematode control, and increasing tolerance to abiotic stresses like drought, salinity, and heavy metals. However, the widespread adoption of fusion protein technology in agriculture will require rigorous field trials, environmental impact assessments, and effective regulatory frameworks to ensure biosafety and public acceptance. With continued research and multidisciplinary collaborations, fusion proteins have the potential to revolutionize crop protection strategies and contribute significantly to global food security. They offer a effective and environmentally friendly alternative to conventional chemical pesticides, aligning with the principles of sustainable agriculture and integrated pest management.

## Data Availability

No datasets were generated or analysed during the current study.
